# The Association of Preoperative Opioid Use with Postdischarge Outcomes: A Cohort Study of the Michigan Surgical Quality Collaborative

**DOI:** 10.1097/SLA.0000000000006265

**Published:** 2024-03-14

**Authors:** Stephan G. Frangakis, Bethany Kavalakatt, Vidhya Gunaseelan, Yenling Lai, Jennifer Waljee, Michael Englesbe, Chad M. Brummett, Mark C. Bicket

**Affiliations:** * Department of Anesthesiology, University of Michigan Medical School, Ann Arbor, MI; † Lake Erie College of Osteopathic Medicine, Bradenton, FL; ‡ Opioid Prescribing and Engagement Network, Institute for Healthcare Innovation and Policy, University of Michigan, Ann Arbor, MI; § Department of Surgery, Section of Plastic Surgery, University of Michigan Medical School, Ann Arbor, MI; ∥ Department of Surgery, Section of Transplantation Surgery, University of Michigan Medical School, Ann Arbor, MI; ¶ Opioid Research Institute, University of Michigan, Ann Arbor, MI

**Keywords:** opioids, surgical outcomes, patient-reported outcomes, preoperative opioid use

## Abstract

**Objective:**

To examine the association of prescription opioid fills over the year before surgery with postoperative outcomes.

**Background:**

Nearly one-third of patients report opioid use in the year preceding surgery, yet an understanding of how opioid exposure influences patient-reported outcomes after surgery remains incomplete. Therefore, this study was designed to test the hypothesis that preoperative opioid exposure may impede recovery in the postoperative period.

**Methods:**

This retrospective cohort study used a statewide clinical registry from 70 hospitals linked to opioid fulfillment data from the state’s prescription drug monitoring program to categorize patients’ preoperative opioid exposure as none (naïve), minimal, intermittent, or chronic. Outcomes were patient-reported pain intensity (primary), as well as 30-day clinical and patient-reported outcomes (secondary).

**Results:**

Compared with opioid-naïve patients, opioid exposure was associated with higher reported pain scores at 30 days after surgery. Predicted probabilities were higher among the opioid exposed versus naive group for reporting moderate pain [43.5% (95% CI: 42.6%–44.4%) vs 39.3% (95% CI: 38.5%–40.1%)] and severe pain [13.% (95% CI: 12.5%–14.0%) vs 10.0% (95% CI: 9.5%–10.5%)], and increasing probability was associated increased opioid exposure for both outcomes. Clinical outcomes (incidence of emergency department visits, readmissions, and reoperation within 30 days) and patient-reported outcomes (reported satisfaction, regret, and quality of life) were also worse with increasing preoperative opioid exposure for most outcomes.

**Conclusions:**

This study is the first to examine the effect of presurgical opioid exposure on both clinical and nonclinical outcomes in a broad cohort of patients and shows that exposure is associated with worse postsurgical outcomes. A key question to be addressed is whether and to what extent opioid tapering before surgery mitigates these risks after surgery.

The burden of the opioid crisis continues to rise, and while the bulk of mortality stems from illicit fentanyl products, prescription opioids remain a consistent contributor to many risks from opioids, including overdoses and deaths. Despite reductions in prescription opioid use since the peak in 2014, an estimated one-third of surgical patients report opioid use in the year preceding surgery, and more than 9 million patients misuse opioids.^1–3^ Preoperative opioid use can occur secondary to underlying conditions and comorbidities that make patients more complex, and, in addition, can itself constitute a risk factor to patients’ health through unwanted drug interactions and adverse physiological changes. Therefore, it is crucial to understand the effects of preoperative opioid use and how these analgesics may increase the overall risks of surgery, anesthesia, and the postoperative period.

Preoperative opioid use, which has been examined in various surgical populations,^4–7^ has traditionally been limited to analyses of health insurance claims. For example, opioid fills before outpatient surgery examined among Medicare claims were associated with 1.68 greater odds of mortality at 90 days.^3^ Although prescription fills are captured in claims data, these investigations can lack insight into patient-reported outcomes and more precise measures, such as actual opioid consumption, pain intensity, and satisfaction ratings. Cohort studies provide some estimates of these patient-centered outcomes, but often rely on smaller sample sizes and lack a broader context of opioid use beyond that reported by patients or captured within the electronic health record of one hospital. Only recently have larger cohort studies examined the immediate surgical and anesthetic complications exacerbated by verified preoperative opioid use, which include readmissions,^8^ and postsurgical opioid use.^9^ In addition, preoperative opioid use has been shown to be associated with an increase in postdischarge adverse opioid-related outcomes.^10^ However, it remains unclear what the association of preoperative opioid use is with postsurgical pain, patient-reported outcomes, and indicators of morbidity, such as emergency room visits, readmissions, and reoperations.

In response to these gaps, we conducted this study to examine the association of prescription opioid use in the year before surgery with postoperative patient-reported outcomes over the 30-day period after discharge from surgery. To do so, we evaluated more than 24,000 patients who underwent one of 9 general and gynecologic surgical procedures using a statewide surgical registry with detailed perioperative data to form the largest and most diverse study on the postoperative outcomes of preoperative opioid use. We then added granular data on prescription opioid fulfillment from the state’s prescription drug monitoring program (PDMP) to assess for preoperative opioid fill up to one year before surgery. We hypothesized that patients who are exposed to opioids in the year before surgery, compared with those who have no exposure in the year before surgery, would have worse patient-reported and clinical outcomes after surgery, and that the level of opioid exposure would be directly associated with worsening postsurgical outcomes.

## METHODS

This was a retrospective cohort study using a statewide clinical registry linked to the state’s PDMP. The Michigan Surgical Quality Collaborative (MSQC) maintains a clinical registry that collects patient demographics, perioperative processes, and 30-day outcomes for patients undergoing surgery in Michigan, as previously described.^11^ Specifically, the MSQC registry captures patient-reported outcomes by surveying patients between 30 and 90 days after the surgery. The 70 participating hospitals receive funding from Blue Cross Blue Shield of Michigan to employ trained data abstractors who use standardized methods to obtain data through a review of the electronic health record specific to each patient. Cases are audited annually for accuracy and reviewed using a sampling algorithm designed to minimize selection bias.^12^ The Institutional Review Board of the University of Michigan classified this study as exempt from review and did not require informed consent as it was an analysis of deidentified data.

Prescription data were obtained using the Michigan Automated Prescription System (MAPS). The MAPS database is Michigan’s PDMP, which tracks all controlled substance fills (schedule 2–5) for residents of Michigan. Within this database, all controlled substance prescription fills, including those for prescription opioids, are uniquely linked to individuals. The purpose of MAPS is to allow prescribers to monitor whether patients have existing controlled substance prescriptions or prescriptions from multiple providers and assess the associated risk before providing a new prescription. Data linkage between patient data from MSQC and prescription data from MAPS was performed based on a state-approved process by an independent third-party data broker that provided encrypted, deidentified data for analysis.

### Study Cohort and Period

We included participants undergoing surgery from January 1, 2017 to October 31, 2019. The study cohort consisted of adult patients ≥18 years who underwent elective, emergent, or urgent surgery in outpatient and inpatient settings. Surgical procedures included laparoscopic appendectomy, laparoscopic cholecystectomy, colectomy (laparoscopic or open), hernia (major or minor), and hysterectomy (laparoscopic, vaginal, or abdominal).

We excluded any patients who died within 30 days of surgery. We excluded non-Michigan residents as MAPS does not capture out-of-state controlled substance fills, so prescription fills would be incomplete for these patients. We also excluded patients who were matched to more than one patient in MAPS due to the inability to uniquely identify a patient and reconcile prescription data. Finally, we excluded patients who were not discharged to home, patients with a length of stay (LOS) >14 days, and patients with missing or incomplete data for outcomes and covariates (with the exception of “unknown race”).

### Explanatory Variables

The key explanatory variable was preoperative opioid exposure, defined as an opioid prescription filled in the 365 days to 31 days before admission to surgery in MAPS. Preoperative opioid exposure was classified using a previous definition into 4 groups with the following mutually exclusive categories based on quantity and duration: (1) naïve, no opioid prescription fills, (2) minimal, ≤1-month fill with <675 oral morphine equivalents (ie, 90 pills of oxycodone 5 mg), (3) intermittent, between 1 month with ≥675 oral morphine equivalents and 8 months filled, and (4) chronic ≥9 months filled.^9^ In addition, to allow for comparison between no opioid exposure and any opioid exposure, preoperative opioid exposure was classified as “yes” for patients with at least one opioid prescription fill, and “no” for patients with no opioid prescription fills in the 365 days to 31 days before surgery.

Demographic data included age, sex, and race/ethnicity. Patient characteristics included American Society of Anesthesiologists physical status classification, obesity (body mass index: >30 kg/m^2^), tobacco use in the year before surgery, and relevant patient comorbidities (cancer, diabetes, chronic obstructive pulmonary disease, and congestive heart failure). Procedure and clinical characteristics included the type of surgical procedure, admission status (inpatient vs outpatient), surgical priority (elective vs urgent/emergent), and LOS in days. Postoperative complications within 30 days of surgery, categorized as any complication or none (yes/no), were also included (Supplemental Methods for list of complications, Supplemental Digital Content 1, http://links.lww.com/SLA/F42).

### Outcomes

The primary outcome of our study was postoperative pain. The survey asks patients to respond to the following question: “Thinking back, how would you rate your pain in the first week after your surgery?” Response options on a 4-point Likert Scale were as follows: no pain (“1”), mild pain (“2”), moderate pain (“3”), and severe pain (“4”).

Secondary outcomes included patient assessment of overall satisfaction, quality of life, and regret of a decision to undergo surgery. Survey questions and possible responses are detailed in the Supplemental Methods (Supplemental Digital Content 1, http://links.lww.com/SLA/F42). Also included in the secondary outcomes were the following as abstracted from the medical record and patient surveys: (1) the incidence of emergency department (ED) visits within the 30 days after surgery, (2) the incidence of hospital readmissions within the 30 days after surgery, and (3) the incidence of reoperations within the 30 days after surgery from MSQC.

### Statistical Analyses

Baseline clinical and demographic data were analyzed for the 4 groups of preoperative opioid exposure using descriptive statistics. Initial comparisons of outcomes were performed using χ^2^ or 1-way analysis of variance, as appropriate. Multilevel ordered logistic regression analysis with a surgeon as the random intercept was performed on the dependent variable of postoperative pain scores (primary outcome) using the 4 groups of opioid exposure in the year before surgery, with the previously mentioned explanatory variables as additional independent variables. In a secondary analysis, a similar multilevel-ordered logistic regression analysis was used with any opioid exposure as the main explanatory variable.

Multilevel logistic regression models adjusting for explanatory variables detailed previously with a surgeon as the random intercept were used to evaluate the association of 4 groups of preoperative opioid exposure with each of the other patient-reported outcomes and clinical outcomes within 30 days. Similar multilevel logistic regression models with a surgeon as the random intercept were used to evaluate the association of any opioid exposure with each of the patient-reported outcomes and clinical outcomes. Based on this, predicted probabilities for opioid exposure were estimated. Finally, sensitivity analysis was performed by reanalysis of data after excluding patients with complications. The significance level for all tests was set at *P* <0.05. Analyses were performed using Stata/SE V.15.1 (StataCorp).

## RESULTS

### Cohort Characteristics

Among 70 participating hospitals, 24,888 patient records met inclusion criteria for analysis (Supplemental Digital Content Table 1, http://links.lww.com/SLA/F43), including 57% females, 83% White non-Hispanic, and an average age of 54.3 years (Table 1). Most patients were opioid-naïve [18,258 (73%)], whereas 3520 (14%) patients had minimal opioid exposure, 2512 (10%) patients had intermittent opioid exposure, and 598 (3%) patients had chronic opioid exposure, based on our previously defined categories.^9^

**TABLE 1:** Patient Characteristics

Characteristic	Overall (N = 24888); n (%)	Naive (N = 18258); n (%)	Minimal (N = 3520); n (%)	Intermittent (N = 2512); n (%)	Chronic (N = 598); n (%)	*P*
Age (yr); mean (SD)*	54.3 (16.4)	54.4 (16.5)	52.5 (16.6)	55.6 (15.5)	57.1 (12.6)	<0.001
Age level						<0.001
18–29	2080 (8.36)	1566 (8.58)	370 (10.51)	136 (5.41)	8 (1.34)	—
30–39	3087 (12.4)	2207 (12.09)	533 (15.14)	293 (11.66)	54 (9.03)	—
40–49	4287 (17.23)	3106 (17.01)	624 (17.73)	452 (17.99)	105 (17.56)	—
50–59	5070 (20.37)	3744 (20.51)	628 (17.84)	533 (21.22)	165 (27.59)	—
60–64	2879 (11.57)	2103 (11.52)	389 (11.05)	296 (11.78)	91 (15.22)	—
≥65	7485 (30.07)	5532 (30.3)	976 (27.73)	802 (31.93)	175 (29.26)	—
Sex						<0.001
Male	10636 (42.74)	8195 (44.88)	1275 (36.22)	928 (36.94)	238 (39.8)	—
Female	14252 (57.26)	10063 (55.12)	2245 (63.78)	1584 (63.06)	360 (60.2)	—
Race/ethnicity						<0.001
White, non-Hispanic	20641 (82.94)	15126 (82.85)	2962 (84.15)	2066 (82.25)	487 (81.44)	—
Black, non-Hispanic	2023 (8.13)	1400 (7.67)	289 (8.21)	264 (10.51)	70 (11.71)	—
Hispanic	616 (2.48)	442 (2.42)	96 (2.73)	68 (2.71)	10 (1.67)	—
Other or unknown	1608 (6.46)	1290 (7.07)	173 (4.91)	114 (4.54)	31 (5.18)	—
ASA						<0.001
Class 1	2188 (8.79)	1848 (10.12)	252 (7.16)	80 (3.18)	8 (1.34)	—
Class 2	14085 (56.59)	10657 (58.37)	1997 (56.73)	1184 (47.13)	247 (41.3)	—
Class 3	8161 (32.79)	5462 (29.92)	1208 (34.32)	1173 (46.7)	318 (53.18)	—
Class 4–5	454 (1.82)	291 (1.59)	63 (1.79)	75 (2.99)	25 (4.18)	—
Comorbidities						
Obese	11630 (46.73)	8211 (44.97)	1808 (51.36)	1321 (52.59)	290 (48.49)	<0.001
Cancer	1497 (6.01)	1066 (5.84)	237 (6.73)	158 (6.29)	36 (6.02)	0.208
Smoker	4865 (19.55)	3267 (17.89)	747 (21.22)	658 (26.19)	193 (32.27)	<0.001
Diabetes	2946 (11.84)	2042 (11.18)	409 (11.62)	387 (15.41)	108 (18.06)	<0.001
COPD	1105 (4.44)	659 (3.61)	161 (4.57)	199 (7.92)	86 (14.38)	<0.001
Congestive heart failure	71 (0.29)	49 (0.27)	4 (0.11)	14 (0.56)	4 (0.67)	0.004
Admission status						<0.001
Inpatient	12192 (48.99)	8830 (48.36)	1728 (49.09)	1321 (52.59)	313 (52.34)	—
Not inpatient	12696 (51.01)	9428 (51.64)	1792 (50.91)	1191 (47.41)	285 (47.66)	—
Urgent/emergent	5817 (23.37)	4470 (24.48)	706 (20.06)	502 (19.98)	139 (23.24)	<0.001
Procedure type						<0.001
Major hernia	1264 (5.08)	827 (4.53)	188 (5.34)	196 (7.8)	53 (8.86)	—
Minor hernia	7421 (29.82)	5655 (30.97)	895 (25.43)	680 (27.07)	191 (31.94)	—
Laparoscopic appendectomy	2458 (9.88)	1988 (10.89)	286 (8.13)	154 (6.13)	30 (5.02)	—
Laparoscopic cholecystectomy	7392 (29.7)	5289 (28.97)	1128 (32.05)	790 (31.45)	185 (30.94)	—
Laparoscopic colectomy	1139 (4.58)	814 (4.46)	178 (5.06)	129 (5.14)	18 (3.01)	—
Open colectomy	698 (2.8)	488 (2.67)	103 (2.93)	85 (3.38)	22 (3.68)	—
Vaginal hysterectomy	1269 (5.1)	933 (5.11)	190 (5.4)	119 (4.74)	27 (4.52)	—
Laparoscopic hysterectomy	2335 (9.38)	1626 (8.91)	416 (11.82)	244 (9.71)	49 (8.19)	—
Total abdominal hysterectomy	912 (3.66)	638 (3.49)	136 (3.86)	115 (4.58)	23 (3.85)	—
LOS (d); median (IQR)	0 (2.0)	0 (2.0)	0 (2.0)	1 (2.0)	1 (2.0)	<0.001
LOS level*						<0.001
LOS=0 d	12696 (51.01)	9428 (51.64)	1792 (50.91)	1191 (47.41)	285 (47.66)	—
0<LOS<=3 d	9772 (39.26)	7107 (38.93)	1411 (40.09)	1011 (40.25)	243 (40.64)	—
3<LOS<=14 d	2420 (9.72)	1723 (9.44)	317 (9.01)	310 (12.34)	70 (11.71)	—
Any complications	709 (2.85)	496 (2.72)	98 (2.78)	96 (3.82)	19 (3.18)	0.018

Preoperative opioid exposure was classified into 4 groups with the following mutually exclusive categories based on quantity and duration: (1) naïve, no opioid prescription fills, (2) minimal, ≤1-month fill with <675 OMEs (ie, 90 pills of oxycodone 5 mg), (3) intermittent, between 1 month with ≥675 OMEs and 8 months filled, and (4) chronic ≥9 months filled. χ^2^ statistical test used for all tests with the exception of mean age (*t* test).

* Age (yr) and LOS level are listed in the table for information only and not used in the models. Only age level and LOS as a continuous variable are used in the models.

ASA indicates American Society of Anesthesiologists; COPD, chronic obstructive pulmonary disease; IQR, interquartile range; OMEs, oral morphine equivalents.

There were notable differences in baseline characteristics among patients belonging to different categories of opioid exposure (Table 1). Patients with increased opioid exposure were more likely to be older, female, Black, and have an American Society of Anesthesiologists classification ≥3. The prevalence of most comorbidities was also higher with increased preoperative opioid exposure; rates of tobacco use, diabetes, and chronic obstructive pulmonary disease all increased with higher opioid exposure. Patients with increased presurgical opioid exposure had more inpatient surgeries and had a longer LOS. Patients with higher opioid exposure were also more likely to undergo more invasive surgical procedures, such as major hernia repair and open colectomy, while less likely to undergo laparoscopic appendectomy.

### Opioid Exposure and Postsurgical Outcomes

**FIGURE 1:**
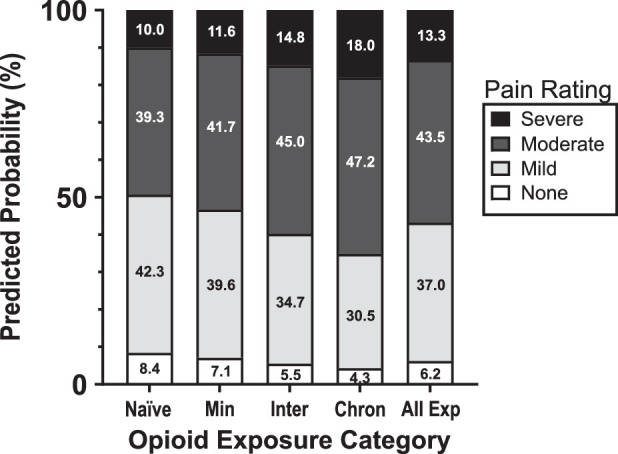
Predicted probability of pain score reporting as a function of opioid exposure category. Naïve, no opioid prescription fills; minimal, ≤ 1-month fill with <675 OMEs (ie, 90 pills of oxycodone 5 mg); intermittent, between 1 month with ≥675 OMEs and 8 months filled; and chronic, ≥9 months filled. All opioid exposure category combines all patients from the minimal, intermittent, and chronic exposure categories. 95% CIs are presented in Supplemental Table 1 (Supplemental Digital Content 2, http://links.lww.com/SLA/F43). OMEs indicates oral morphine equivalents.

We observed a dose-dependent effect of opioid exposure on 30-day pain intensity, patient-reported outcomes, and clinical outcomes. In adjusted models, patients with increasing levels of opioid exposure had higher predicted probabilities of reporting worse pain scores (moderate pain and severe pain), and lower probabilities of reporting better pain scores (no pain or mild pain) (Fig. 1, Supplemental Digital Content Table 2, http://links.lww.com/SLA/F43). As the degree of preoperative opioid exposure increased, this outcome was more pronounced.

**FIGURE 2:**
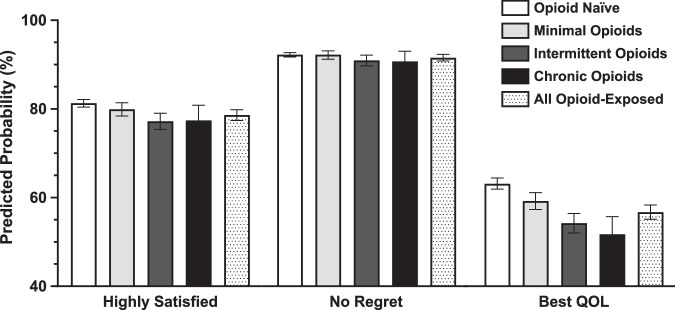
Predicted probability of reporting each patient-reported outcome as a function of opioid exposure category.

**FIGURE 3:**
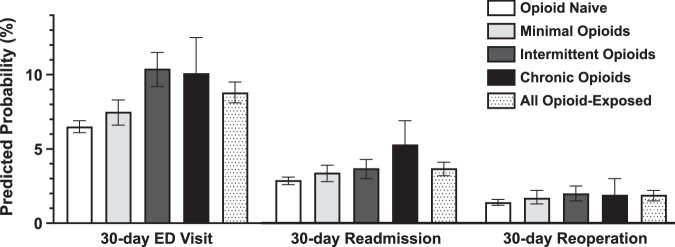
Predicted probability of experiencing each clinical outcome as a function of opioid exposure category, in the first 30 days after surgery.

Adjusted models also showed a lower predicted probability of experiencing the best patient-reported outcomes (reporting “highly satisfied” or “best possible quality of life”) with increasing levels of opioid exposure (Fig. 2, Supplemental Digital Content Table 3, http://links.lww.com/SLA/F43). There was no significant difference in the predicted probability of reporting “no regret” with increasing opioid use, or when comparing patients with any opioid exposure to opioid naïve patients. We found that for clinical outcomes as well, patients with increasing levels of opioid exposure had worse outcomes, with higher predicted probabilities of experiencing an ED visit, readmission, and reoperation (Fig. 3, Supplemental Digital Content Table 3, http://links.lww.com/SLA/F43).

The adjusted odds ratios (aORs) of each exposure group relative to opioid-naïve for pain intensity and patient-reported outcomes are shown in Supplemental Table 4 (Supplemental Digital Content 2, http://links.lww.com/SLA/F43) and Supplemental Table 5 (Supplemental Digital Content 2, http://links.lww.com/SLA/F43), respectively. The aORs of each exposure group for clinical outcomes are shown in Table 2. aORs for the combined group of all opioid exposed versus opioid-naïve patients for pain, patient-reported, and clinical outcomes are shown in Supplemental Table 6 (Supplemental Digital Content 2, http://links.lww.com/SLA/F43). These data were consistent with the predicted probabilities and showed worse overall outcomes with increasing levels of preoperative opioid exposure. These associations remained after sensitivity analyses where patients with any complications were excluded (Supplemental Digital Content Tables 7–10, http://links.lww.com/SLA/F43).

**TABLE 2:** aORs of Opioid Exposure, Demographic, and Procedural Factors from the Multilevel Ordered Logistic Regression Model for Clinical Outcomes

	30 d ED Visit				30 d Readmission				30 d Reoperation			
Characteristics	aOR	95% CI Low	95% CI High	*P*	aOR	95% CI Low	95% CI High	*P*	aOR	95% CI Low	95% CI High	*P*
Preoperative opioid exposure (naïve as reference)												
Minimal	1.17	1.02	1.35	0.024	1.22	0.97	1.52	0.083	1.26	0.94	1.69	0.122
Intermittent	1.69	1.47	1.95	<0.001	1.34	1.06	1.69	0.014	1.47	1.08	2.00	0.014
Chronic	1.65	1.26	2.17	<0.001	2.13	1.46	3.11	<0.001	1.42	0.79	2.55	0.242
Age (age 18–29 as reference)												
30–39	0.83	0.69	1.00	0.050	0.90	0.62	1.31	0.580	2.36	1.26	4.42	0.007
40–49	0.62	0.51	0.74	<0.001	0.79	0.55	1.13	0.202	2.10	1.13	3.89	0.019
50–59	0.45	0.37	0.54	<0.001	0.76	0.54	1.09	0.135	1.53	0.82	2.85	0.180
60–64	0.43	0.34	0.54	<0.001	0.86	0.59	1.27	0.454	1.65	0.86	3.16	0.130
≥65	0.44	0.36	0.53	<0.001	1.07	0.76	1.51	0.708	1.35	0.72	2.52	0.345
Sex; Male (female as reference)	0.86	0.76	0.97	0.017	1.13	0.94	1.36	0.185	0.99	0.77	1.27	0.937
Race/ethnicity (White, non-Hispanic as reference)												
Black, non-Hispanic	1.18	1.00	1.40	0.048	1.32	1.01	1.72	0.040	0.62	0.40	0.97	0.037
Hispanic	1.24	0.94	1.64	0.122	1.19	0.74	1.92	0.483	1.20	0.64	2.25	0.563
Other or unknown	0.69	0.54	0.88	0.003	1.00	0.72	1.39	0.986	1.01	0.66	1.55	0.968
ASA classification (1 as reference)												
Class 2	1.37	1.12	1.68	0.002	1.19	0.81	1.73	0.381	0.78	0.50	1.23	0.286
Class 3	1.72	1.37	2.16	<0.001	1.60	1.06	2.39	0.024	1.12	0.69	1.83	0.635
Class 4–5	1.99	1.33	2.96	0.001	2.45	1.42	4.23	0.001	0.67	0.29	1.52	0.338
Comorbidities												
Obese	0.96	0.87	1.07	0.488	0.92	0.78	1.08	0.308	0.95	0.76	1.19	0.680
Cancer	0.76	0.59	0.97	0.029	1.16	0.86	1.56	0.335	3.58	2.59	4.95	<0.001
Smoker	1.25	1.11	1.40	<0.001	1.38	1.14	1.67	0.001	1.37	1.06	1.78	0.015
Diabetes	1.06	0.91	1.24	0.461	1.11	0.89	1.38	0.352	0.90	0.66	1.24	0.529
COPD	1.38	1.11	1.72	0.004	1.32	0.98	1.79	0.069	1.34	0.89	2.01	0.159
Congestive heart failure	0.95	0.40	2.27	0.916	2.69	1.27	5.70	0.010	0.45	0.06	3.49	0.447
Inpatient admission (not inpatient as reference)	1.13	0.97	1.32	0.130	1.32	1.03	1.69	0.027	0.93	0.66	1.32	0.686
Surgical priority: urgent/emergent	1.15	0.96	1.37	0.139	0.87	0.67	1.13	0.284	1.41	0.99	2.00	0.055
Procedure type (major hernia as reference)												
Minor hernia	0.97	0.76	1.25	0.818	0.60	0.41	0.88	0.009	0.89	0.54	1.46	0.641
Laparoscopic appendectomy	0.97	0.72	1.31	0.844	1.00	0.64	1.57	0.992	0.48	0.26	0.91	0.024
Laparoscopic cholecystectomy	1.04	0.81	1.32	0.771	1.33	0.94	1.89	0.107	0.72	0.45	1.16	0.180
Laparoscopic colectomy	0.95	0.66	1.36	0.776	0.73	0.46	1.17	0.190	1.21	0.69	2.12	0.503
Open colectomy	1.52	1.04	2.21	0.029	0.95	0.59	1.52	0.816	0.68	0.37	1.24	0.208
Vaginal hysterectomy	0.76	0.54	1.07	0.122	0.73	0.44	1.21	0.221	0.84	0.43	1.64	0.616
Laparoscopic hysterectomy	1.13	0.85	1.50	0.390	0.70	0.44	1.11	0.126	0.46	0.24	0.87	0.017
Total abdominal hysterectomy	1.03	0.73	1.46	0.860	0.76	0.46	1.25	0.275	0.59	0.29	1.16	0.126
LOS	0.95	0.91	0.99	0.013	1.07	1.02	1.12	0.004	1.14	1.08	1.21	<0.001
Any complications	4.42	3.64	5.37	<0.001	22.34	18.26	27.34	<0.001	4.96	3.64	6.76	<0.001
	Predicted Probabilities (%)	95% CI Low (%)	95% CI High (%)	*P*	Predicted Probabilities (%)	95% CI Low (%)	95% CI High (%)	*P*	Predicted Probabilities (%)	95% CI Low (%)	95% CI High (%)	*P*
Preoperative opioid exposure	—	—	—	<0.001	—	—	—	<0.001	—	—	—	0.049
Naïve	6.5	6.1	6.8	—	2.9	2.6	3.1	—	1.4	1.2	1.6	—
Minimal	7.5	6.6	8.3	—	3.4	2.8	3.9	—	1.7	1.3	2.2	—
Intermittent	10.4	9.2	11.5	—	3.7	3.0	4.3	—	2.0	1.5	2.5	—
Chronic	10.1	7.8	12.5	—	5.3	3.8	6.9	—	1.9	0.9	3.0	—

ASA indicates American Society of Anesthesiologists; COPD, chronic obstructive pulmonary disease.

### Demographic/Procedural Factors and Postsurgical Outcomes

We observed several patient demographic and procedural factors that were independent of opioid exposure but were also significantly associated with postsurgical outcomes. Decreased age, female sex, and Black race were associated with higher aOR for reporting higher pain intensity (Supplemental Digital Content Table 4, http://links.lww.com/SLA/F43). Several patient and demographic factors were also found to be significantly associated with patient-reported (Supplemental Digital Content Table 5, http://links.lww.com/SLA/F43) and clinical outcomes (Table 2). Sensitivity analyses are shown in Supplemental Tables 7 to 9 (Supplemental Digital Content 2, http://links.lww.com/SLA/F43).

## DISCUSSION

This population-based cohort study used registry data linked to a state PDMP to examine the association of exposure to prescription opioids in the year before surgery with outcomes after discharge from surgery. We found that the level of preoperative opioid exposure was significantly associated with the postsurgical outcomes experienced by patients, even after adjusting for all patient, clinical, and procedural factors. Increasing levels of opioids were associated with higher pain scores, worse patient-reported outcomes, and overall worse clinical outcomes. Most notably, as patients’ level of opioid exposure rose, the percentage of patients reporting their pain as “moderate” or “severe” rose by 4% to 6% for each exposure level. This suggests that presurgical opioid use has a strong association with postsurgical pain and raises interest in answering questions as to whether interventions that lead to reductions or tapering in opioid use before surgery may lead to improvements in the ability to manage postsurgical pain.

Clinical outcomes 30 days after surgery were also worse in opioid-exposed patients. Patients with any presurgical opioid exposure were 1.4 times more likely to experience a postoperative ED visit than patients with opioid naïve, and those in the intermittent and chronic categories were even more likely to have an ED visit than those in the minimal category. The likelihood of hospital readmission within 30 days was also greater in opioid-exposed patients, with the odds increasing with each subsequent exposure category (naïve < minimal < intermittent < chronic). Studies have shown the importance of perioperative factors in influencing postsurgical readmission, which has been difficult to predict.^13,14^ Here we demonstrate that preoperative opioid exposure is significantly associated with the likelihood of patient readmission. There was also an increased likelihood of reoperation, though this was only significant for those patients in the intermittent group. This is likely due to the much lower overall incidence of reoperation, with a predicted probability of only 1% to 2% in each group.

In a recent study, nearly a quarter (23.1%) of surgical patients reported preoperative opioid use,^15^ similar to the 27% found in our cohort. This prevalence underscores the importance of understanding the effects of preoperative opioids. A recent smaller study in an orthopedic cohort demonstrated that current opioid use was associated with postoperative pain.^16^ Larger cohort studies have examined the immediate surgical and anesthetic complications exacerbated by verified preoperative opioid use, which include readmissions, reoperations, and continued postoperative opioid use.^8–10,17–21^ Here we utilized a large and surgically diverse patient population, evaluating both clinical and patient-reported outcomes within the same cohort.

### Clinical Implications

Although this study suggests that reducing patients’ opioid use before surgery may lead to improvements in postsurgical pain and other outcomes, this must be weighed against the known risks of opioid tapering. Current literature suggests an association between opioid tapering/discontinuation and increased risks for opioid overdose and suicide, though this literature is from nonsurgical cohorts.^22,23^ Tapering of opioids is shown to have a lower risk profile than abrupt discontinuation, especially in patients receiving a stable long-term opioid dosage without evidence of misuse. Thus, reduction in presurgical opioid use has the potential to reduce adverse postsurgical outcomes but should be applied in the appropriate patient population.

### Limitations

This study does have several limitations. First, the associations that were found are not presented as being causal, though the strength of inferences from the data appear robust to suggest that there is an increased likelihood of poorer outcomes as presurgical opioid exposure increases. Second, there is a possibility of unmeasured confounding variables influencing relationships in the analysis, given the absence of preoperative pain scores and other relevant covariates, though we sought to mitigate confounding by adjusting models to account for multiple relevant patient, clinical, and procedural variables. Third, opioid exposure data may differ from actual opioid consumption. However, prior studies have shown that state PDMP data are reliable in detecting perioperative opioid fills,^24^ our previous examination of opioid consumption and prescription in this population suggests that this may not be an issue,^9^ and our prior work shows that consumption is correlated with prescribing. In addition, we took full advantage of databases that link prescription drug monitoring programs (PDMPs) and pharmacy dispensation databases to provide a detailed view of patients’ prescription fills in the year before surgery. These databases have been shown to be reliable in identifying opioid fulfillment in the perioperative period.^24^ We were able to obtain prescriptions, electronic health records, and survey data from tens of thousands of patients and link each data set. Finally, this study did not evaluate the reasons and/or indications for presurgical opioid exposure, which have been shown to be associated with postsurgical outcomes. Presurgical pain intensity, though shown to influence postsurgical pain, was not a variable in this analysis.

## CONCLUSIONS

This study demonstrates that, in a large and diverse cohort, preoperative exposure to opioids was significantly associated with worse postsurgical patient-reported pain, as well as many other outcomes and health care utilization after surgery. These included increased pain intensity, negative clinical outcomes, and unfavorable patient-reported outcomes. In addition, we found that higher levels of preoperative opioid consumption were associated with worse outcomes in a dose-dependent manner. Preoperative opioid exposure is, therefore, a significant, but potentially modifiable, factor associated with postsurgical outcomes. A patient’s opioid use should be identified as a risk factor for these worse surgical outcomes, while a key question to be addressed is whether and to what extent opioid tapering before surgery mitigates these risks after surgery.

## Supplementary Material

## References

[1] Katzman C Harker EC Ahmed R . The association between preoperative opioid exposure and prolonged postoperative use. Ann Surg. 2021;274:e410–e416. 32427764 10.1097/SLA.0000000000003723

[2] Vu JV Cron DC Lee JS . Classifying preoperative opioid use for surgical care. Ann Surg. 2020;271:1080–1086. 30601256 10.1097/SLA.0000000000003109 PMC7092502

[3] Agarwal S Shah A Gunaseelan V . New persistent opioid use after surgery in patients with a history of remote opioid use. Surgery. 2022;171:1635–1641. 34895768 10.1016/j.surg.2021.11.008

[4] Bonner BE Castillo TN Fitz DW . Preoperative opioid use negatively affects patient-reported outcomes after primary total hip arthroplasty. J Am Acad Orthop Surg. 2019;27:e1016–e1020. 30829899 10.5435/JAAOS-D-18-00658

[5] Cozowicz C Olson A Poeran J . Opioid prescription levels and postoperative outcomes in orthopedic surgery. Pain. 2017;158:2422–2430. 28891865 10.1097/j.pain.0000000000001047

[6] Subramanian MP Sahrmann JM Nickel KB . Assessment of preoperative opioid use prevalence and clinical outcomes in pulmonary resection. Ann Thorac Surg. 2021;111:1849–1857. 33011165 10.1016/j.athoracsur.2020.07.043 PMC8227806

[7] Ingall E Klemt C Melnic CM . Impact of preoperative opioid use on patient-reported outcomes after revision total knee arthroplasty: a propensity matched analysis. J Knee Surg. 2023;36:115–20. 33992033 10.1055/s-0041-1729966

[8] Tang R Santosa KB Vu JV . Preoperative opioid use and readmissions following surgery. Ann Surg. 2022;275:e99–e106. 32187028 10.1097/SLA.0000000000003827 PMC7935087

[9] Bicket MC Gunaseelan V Lagisetty P . Association of opioid exposure before surgery with opioid consumption after surgery. Reg Anesth Pain Med. 2022;47:346–352. 35241626 10.1136/rapm-2021-103388 PMC9035103

[10] Kim K Biskupiak JE Babin JL . Positive association between peri-surgical opioid exposure and post-discharge opioid-related outcomes. Healthcare (Basel). 2022;11:115. 36611576 10.3390/healthcare11010115 PMC9819163

[11] Campbell DA Jr Englesbe MJ Kubus JJ . Accelerating the pace of surgical quality improvement: the power of hospital collaboration. Arch Surg. 2010;145:985–991. 20956768 10.1001/archsurg.2010.220

[12] Healy MA Regenbogen SE Kanters AE . Surgeon variation in complications with minimally invasive and open colectomy: results from the Michigan surgical quality collaborative. JAMA Surg. 2017;152:860–867. 28614551 10.1001/jamasurg.2017.1527 PMC5710462

[13] Morris MS Graham LA Richman JS . Postoperative 30-day readmission: time to focus on what happens outside the hospital. Ann Surg. 2016;264:621–631. 27355263 10.1097/SLA.0000000000001855

[14] Hernandez-Boussard T Graham LA Desai K . The fifth vital sign: postoperative pain predicts 30-day readmissions and subsequent emergency department visits. Ann Surg. 2017;266:516–524. 28657940 10.1097/SLA.0000000000002372 PMC6530481

[15] Hilliard PE Waljee J Moser S . Prevalence of preoperative opioid use and characteristics associated with opioid use among patients presenting for surgery. JAMA Surg. 2018;153:929–937. 29998303 10.1001/jamasurg.2018.2102 PMC6233789

[16] Armstrong AD Hassenbein SE Black S . and o.b.o.t.I.P. Team , Risk factors for increased postoperative pain and recommended orderset for postoperative analgesic usage. Clin J Pain, 2020;36: 845-851. 32889819 10.1097/AJP.0000000000000876 PMC7671821

[17] Blevins Peratikos M Weeks HL Pisansky AJB . Effect of preoperative opioid use on adverse outcomes, medical spending, and persistent opioid use following elective total joint arthroplasty in the United States: a large retrospective cohort study of administrative claims data. Pain Med. 2020;21:521–531. 31120529 10.1093/pm/pnz083 PMC7060398

[18] Jain N Brock JL Malik AT . Prediction of complications, readmission, and revision surgery based on duration of preoperative opioid use: analysis of major joint replacement and lumbar fusion. J Bone Joint Surg Am. 2019;101:384–391. 30845032 10.2106/JBJS.18.00502

[19] Waljee JF Cron DC Steiger RM . Effect of preoperative opioid exposure on healthcare utilization and expenditures following elective abdominal surgery. Ann Surg. 2017;265:715–721. 28151795 10.1097/SLA.0000000000002117 PMC5767099

[20] Wilson JM Farley KX Bradbury TL . Preoperative opioid use is a risk factor for complication and increased healthcare utilization following revision total knee arthroplasty. Knee. 2020;27:1121–1127. 32711872 10.1016/j.knee.2020.05.013

[21] Wilson JM Farley KX Gottschalk MB . Preoperative opioid use is an independent risk factor for complication, revision, and increased health care utilization following primary total shoulder arthroplasty. J Shoulder Elbow Surg. 2021;30:1025–1033. 32853788 10.1016/j.jse.2020.08.007

[22] Larochelle MR Lodi S Yan S . Comparative effectiveness of opioid tapering or abrupt discontinuation vs no dosage change for opioid overdose or suicide for patients receiving stable long-term opioid therapy. JAMA Netw Open. 2022;5:e2226523. 35960518 10.1001/jamanetworkopen.2022.26523 PMC9375167

[23] Bohnert ASB Ilgen MA . Understanding links among opioid use, overdose, and suicide. N Engl J Med. 2019;380:71–79. 30601750 10.1056/NEJMra1802148

[24] Fernandez AC Bohnert A Gunaseelan V . Identifying persistent opioid use after surgery: the reliability of pharmacy dispensation databases. Ann Surg. 2023;278:e20–e26. 35815891 10.1097/SLA.0000000000005529 PMC9832314

