# Supplementary material for: The Association of Preoperative Opioid Use with Postdischarge Outcomes: A Cohort Study of the Michigan Surgical Quality Collaborative
Source: Ann Surg. 2024 Mar 14;282(2):234–41. doi: 10.1097/SLA.0000000000006265 (PMC11399320; doi:10.1097/SLA.0000000000006265)
Supplement: Supplementary file 1 [file sla-282-234-s001.docx]

# Supplemental Methods

The various complications considered were: anastomotic leak, cardiac arrest, Clostridium difficile infection, deep incisional surgical site infection, deep vein thrombosis, myocardial infarction, organ space surgical site infection, pneumonia, pulmonary embolism, sepsis, severe sepsis/septic shock, stroke, superficial incisional surgical site infection, transfusions within 72 hours postop, and urinary tract infection.

Regret undergoing surgery was reported from a survey given between postoperative days 30 and 90 which uses the 5-point Likert Scale Regret Rating. The survey asked patients to respond to the following question: “Do you regret your decision to undergo surgery?” The options on the Likert Scale Regret Rating are as follows: 1= extremely regret surgery to 5= absolutely no regret. Patient reported quality of life (QOL) was obtained from a survey given between postoperative days 30 and 90 which uses the 5-point Likert QOL Rating. The survey asked patients to respond to the following question: “In general, what do you say your quality of life is?” The options on the Likert Scale QOL Rating are as follows: 1= worst possible to 5= best possible. Patient satisfaction was obtained from a survey given between postoperative days 30 and 90 using the 10-point Likert Satisfaction Rating. The survey asked patients to respond to the following question: “Overall, how would you rate your satisfaction with your experience after surgery?” The options on the Likert Scale Satisfaction Rating are as follows: 0= extremely dissatisfied to 10= extremely satisfied.

Quality of life outcomes were dichotomized into “absolute best quality of life” for a score of 5 and “less than best quality of life” for scores of 1-4. Patient satisfaction was dichotomized into “highly satisfied” for scores of 9-10 and “not highly satisfied” for scores of 1-8. Regret to undergo surgery was dichotomized into “absolutely no regret” for a score of 5 and “some regret” for scores of 1-4.
